# Detrimental Effects of Helium Ion Irradiation on Cognitive Performance and Cortical Levels of MAP-2 in B6D2F1 Mice

**DOI:** 10.3390/ijms19041247

**Published:** 2018-04-20

**Authors:** Jacob Raber, Eileen Ruth S. Torres, Tunde Akinyeke, Joanne Lee, Sydney J. Weber Boutros, Mitchell S. Turker, Amy Kronenberg

**Affiliations:** 1 Department of Behavioral Neuroscience, Oregon Health and Science University, Portland, OR 97239, USA; torreei@ohsu.edu (E.R.S.T.); akinyeke@ohsu.edu (T.A.); joanneslee3@gmail.com (J.L.); webesy@ohsu.edu (S.J.W.B.); 2 Departments of Neurology and Radiation Medicine, Division of Neuroscience ONPRC, Oregon Health and Science University, Portland, OR 97239, USA; 3 Oregon Institute of Occupational Health Sciences and Department of Molecular and Medical Genetics, Oregon Health and Science University, Portland, OR 97239, USA; turkerm@ohsu.edu; 4 Biological Systems and Engineering Division, Lawrence Berkeley National Laboratory, Berkeley, CA 94720, USA; a_kronenberg@lbl.gov

**Keywords:** object recognition, passive avoidance, fear conditioning, MAP-2, galactic cosmic radiation, helium ions

## Abstract

The space radiation environment includes helium (^4^He) ions that may impact brain function. As little is known about the effects of exposures to ^4^He ions on the brain, we assessed the behavioral and cognitive performance of C57BL/6J × DBA2/J F1 (B6D2F1) mice three months following irradiation with ^4^He ions (250 MeV/n; linear energy transfer (LET) = 1.6 keV/μm; 0, 21, 42 or 168 cGy). Sham-irradiated mice and mice irradiated with 21 or 168 cGy showed novel object recognition, but mice irradiated with 42 cGy did not. In the passive avoidance test, mice received a slight foot shock in a dark compartment, and latency to re-enter that compartment was assessed 24 h later. Sham-irradiated mice and mice irradiated with 21 or 42 cGy showed a higher latency on Day 2 than Day 1, but the latency to enter the dark compartment in mice irradiated with 168 cGy was comparable on both days. ^4^He ion irradiation, at 42 and 168 cGy, reduced the levels of the dendritic marker microtubule-associated protein-2 (MAP-2) in the cortex. There was an effect of radiation on apolipoprotein E (apoE) levels in the hippocampus and cortex, with higher apoE levels in mice irradiated at 42 cGy than 168 cGy and a trend towards higher apoE levels in mice irradiated at 21 than 168 cGy. In addition, in the hippocampus, there was a trend towards a negative correlation between MAP-2 and apoE levels. While reduced levels of MAP-2 in the cortex might have contributed to the altered performance in the passive avoidance test, it does not seem sufficient to do so. The higher hippocampal and cortical apoE levels in mice irradiated at 42 than 168 cGy might have served as a compensatory protective response preserving their passive avoidance memory. Thus, there were no alterations in behavioral performance in the open filed or depressive-like behavior in the forced swim test, while cognitive impairments were seen in the object recognition and passive avoidance tests, but not in the contextual or cued fear conditioning tests. Taken together, the results indicate that some aspects of cognitive performance are altered in male mice exposed to ^4^He ions, but that the response is task-dependent. Furthermore, the sensitive doses can vary within each task in a non-linear fashion. This highlights the importance of assessing the cognitive and behavioral effects of charged particle exposure with a variety of assays and at multiple doses, given the possibility that lower doses may be more damaging due to the absence of induced compensatory mechanisms at higher doses.

## 1. Introduction

The radiation environment in deep space contains all of the naturally-occurring energetic charged particles, including helium ions (^4^He). Helium ions are technically the same as α particles, (i.e., a helium atom without its electrons), but the energies of most ^4^He ions in the galactic cosmic radiation (GCR) are much higher than what is found amongst α particles, resulting from the decay of terrestrial elements (e.g., radon). As a result, the space radiation literature typically refers to “^4^He ions” to distinguish them from lower energy “α particles”. Notably, the energy deposition characteristics of high energy ^4^He ions, which can travel long distances in tissue and are sparsely ionizing until they have slowed near the very end of their particle track, are very different from those associated with α decay from, e.g., radon, which starts off with much lower velocity and is quite densely ionizing over much of its very short track in tissue-like materials. 

Exposure to high energy ^4^He ions may pose a significant risk to the central nervous system during and following missions. Helium ions are the second most abundant charged particle in the GCR, comprising 14% of the GCR. Helium ions in GCR can have energies ranging from tens of MeV/n up to 100 GeV/n, with the highest abundance in interstellar space from 100 to 200 MeV/n to a few GeV/n [1]. Helium ions are also found among the GCR spectrum in low Earth orbit, but the exposures from both GCR and solar particle events (SPE) at the ISS for missions of six months indicated average physical doses of 28.9 ± 4.9 mGy, or 2.89 + 0.0.49 cGy [1]. Measurements taken in transit to Mars by the Radiation Assessment Detector indicated average daily exposures behind the shielding around the detector package of 481 ± 80 μGy/day from the silicon detector, or 461 ± 92 μGy/day from a plastic scintillator detector, with only about 4% of the dose attributed to particles emanating from SPE [2]. With an average mission duration of 180 days for transit to Mars, a similar period of time to return and additional dose from time spent on the Martian surface [3], it is expected that the physical dose deposited from such a Mars mission would be substantially higher than what astronauts have received on six-month excursions on the ISS, approaching or exceeding NASA’s current dose limits for missions in low Earth orbit [2]. 

The effects of other space radiation-relevant charged particles such as ^56^Fe [4,5,6,7,8,9,10,11], ^28^Si [12,13,14], ^48^Ti [15], ^40^Ca [16] and ^16^O [17] ions on the brain have been studied using ground-based accelerators as the source of the ions of interest. Brain injury has been studied with MRI and PET following irradiation of the rabbit brain with clinically-relevant doses of 11.5 and 23.1 Gy of ^4^He ions (230 MeV/n) [18]. ^4^He ions, given as five equal fractions of 10–16 Gy at 232 MeV/n) have been used in patients with uveal melanoma [19] and in in vivo tumor systems [20]. In contrast to these high dose, clinically-relevant exposures, the effects of more modest exposures of ^4^He ions on the brain are less well understood in rats [21], and to the best of our knowledge, they have not been reported yet in mice.

Early studies on the effects of ^4^He ions in rats indicated there was a significant increase in body temperature 10 min following exposure to ^4^He (0.5 Gy, 165 MeV/n) [22]. In addition, ^4^He exposure (0.5 Gy, 165 MeV/n) caused rats to reduce sucrose intake 24 h after exposure in a dose-dependent manner (ED_50_ of 121 cGy) [23]. A more recent study with very low dose, head-only exposures of rats to ^4^He ions (1000 MeV/n) reported increased measures of anxiety in the elevated plus maze and reduced, but did not eliminate, preference for an object in a novel location 4 h after training at doses between 0.1 and 5 cGy 1–5 months after exposure [24]. However, none of the doses reduced novel object recognition of rats 24 h after training, and an increased preference relative to controls for the novel object was observed at 0.5, 1 and 5 cGy 1–5 months after exposure [24].

The environmental conditions astronauts experience during space missions, especially long space missions, include not only ionizing radiation, but also psychological and physical stressors [25]. Exposure to space radiation might cause not only cognitive, but also behavioral alterations and modulate the response of astronauts to these stressors. Therefore, evaluating behavioral performance, including the response to controlled environmental emotional stressors is also important. Behavioral measures pertinent to astronauts during space missions include measures of anxiety, depression and circadian activity [25]. These behavioral measures can be assessed, under controlled environmental conditions in translational tests in animal models.

With regard to cognitive performance, object recognition [26,27] and contextual and cued fear learning and memory [7,12,13,28] were sensitive to detect the effects of certain high energy charged particle exposures of relevance to space flight using a ground-based accelerator as the source of these ions. Some types of accelerator-derived charged particles also affected response to a novel environment. Baseline activity in a novel environment was reduced in five-month-old C57BL/6J mice three months following ^56^Fe ion irradiation (600 MeV/n, 174 keV/µm, 0.5 Gy) [4]. While the object recognition and fear conditioning tests detected effects of charged particle exposures, the relationship between the dose of each ion, its linear energy transfer (LET, a measure of the density of energy deposited in a local area) and cognitive performance on these tests is complex [12,29]. As most mouse radiation studies have been performed using C57Bl6/J mice and genetic factors are anticipated to modulate the radiation response, it is important to assess radiation effects in other genetic backgrounds, such as the B6D2F1 background used in the current study.

Microtubule-associated protein 2 (MAP-2) is a dendritic protein important for stabilizing microtubuli and dendritic plasticity. MAP-2 is required for dendrite elongation [30]. MAP-2 was shown to be a sensitive marker for age-related changes in rodents [31,32] and nonhuman primates [33] and can also be affected by irradiation. When one-month-old C57BL/6J mice were trained for contextual fear conditioning, followed by irradiation (X-rays, whole body, 4 Gy) one day later and extinction assessed over eight days starting 14 days after training, MAP-2 levels in the hippocampus were increased in mice that received five shocks during training [34]. In mice expressing human apolipoprotein E3 under the control of the mouse apoE promoter, MAP-2 immunoreactivity in the hippocampus, cortex and amygdala was increased three months following ^137^Cs irradiation (head only, 10 Gy) at two months of age [35].

Inhibited transcription of brain-derived neurotrophic factor (BDNF) has been implicated after clinically-relevant radiation doses to the whole brain that produced cognitive injury in rats (30 Gy; 4-MV electrons) and mice (10 Gy; 6-MV photons) [36,37]. The effect of microglia, resident macrophages in the brain, on learning-dependent synapse formation involves the release of BDNF [38]. This release is important for cognitive performance. Activation of microglia, important in neuroinflammation, triggers the release of BDNF, which in turn induces the proliferation and prolonged activation of microglia [39,40,41]. CD68 (macrosialin), a lysosome-associated membrane glycoprotein, is a marker of activated microglia [42,43], and CD68 levels were increased in the mouse brain following a moderate (2 Gy) whole-body exposure to gamma rays [44,45]. An increase in activated microglia, assessed as immunoreactive ED-1 cells, was also reported in the medial prefrontal cortex of male transgenic (Thy1-EGFP) MJrsJ mice 15 and 20 weeks following ^16^O (600 MeV/n; 5 or 30 cGy) and 48Ti (600 MeV/n; 5 or 30 cGy) ion irradiation at six months of age [46].

Apolipoprotein E (apoE) plays a role in the transport and metabolism of lipids. In the brain, apoE plays a role in neuronal repair following injury. ApoE might modulate the effects of space irradiation on the brain. Mice deficient in apoE are more susceptible than wild-type mice to the effects of ^56^Fe irradiation on cognition [47], and the effects of ^56^Fe irradiation on cognition at 13 months after irradiation are dependent on the apoE isoform [5,48]. Finally, in mice expressing apolipoprotein E3, there is a trend towards increased brain levels of apoE following ^56^Fe ion irradiation [5].

In the present study, we assessed the behavioral and cognitive performance of B6D2F1 mice three months following exposure to ^4^He ions at four to six months of age (mouse ages pertinent to the age of most astronauts) and whether behavioral and cognitive performance was associated with alterations of MAP-2, CD68, BDNF and apoE levels in the cortex and hippocampus of these mice. 

## 2. Results

### 2.1. Behavioral Performance in the Open Field and Object Recognition

**Figure 1:**
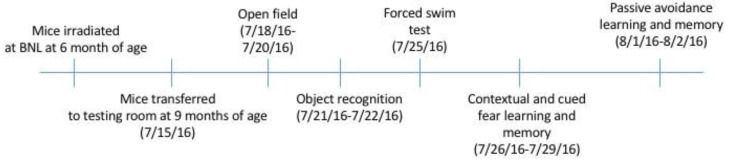
Behavioral testing schedule. The numbers indicated reflect dates and illustrate the time intervals between the distinct tests. The first behavioral test was performed three months following irradiation or sham-irradiation. BNL, Brookhaven National Laboratory.

**Figure 2:**
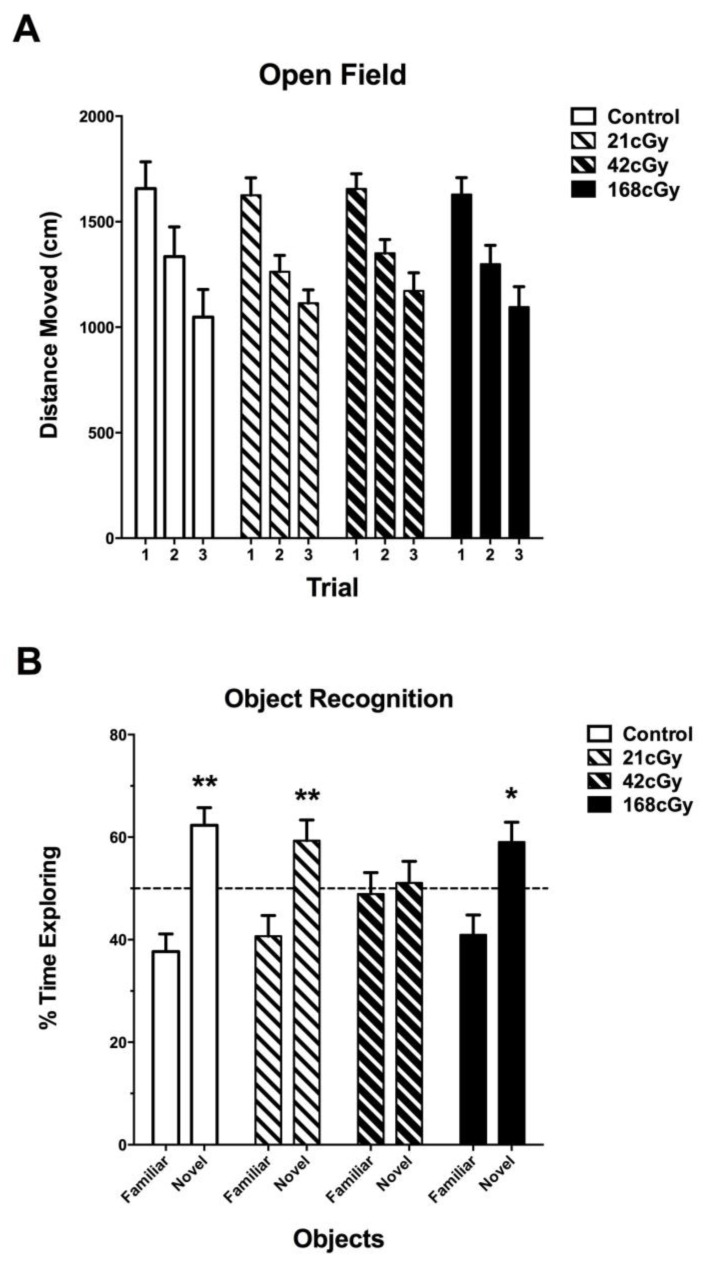
Behavioral performance in the open field and object recognition. (**A**) Habituation to the open field. All mice habituated to the open field (effect of trial, *p* < 0.001), but there was no effect of irradiation. (**B**) Object recognition. Sham-irradiated mice and mice irradiated with 21 cGy or 168 cGy showed object recognition and spent more time exploring the novel object than the familiar object, but mice irradiated with 42 cGy showed impaired object recognition. Control: *n* = 13 mice; 21 cGy: *n* = 23 mice; 42 cGy: *n* = 20 mice; 168 cGy: *n* = 16 mice. * *p* < 0.05 versus familiar object; ** *p* < 0.01 versus familiar object.

Animals were irradiated and behaviorally tested according to Figure 1. Based on observations by the researchers and animal staff, the ^4^He ion exposures were well tolerated by the animals, and no obvious adverse effects were observed during the post-irradiation follow-up and testing periods. All mice habituated to the open field (effect of trial, *p* < 0.001), but there was no effect of irradiation (Figure 2A). As there was an effect of sex (*F*(1,64) = 5.438, *p* = 0.023), we also analyzed the female and male data separately. There was no effect of irradiation in females (*p* = 0.552) or males (*p* = 0.498), and this sex effect seemed driven by the higher activity of females than males.

When anxiety levels were assessed by analyzing the percent of time spent in the center of the open field, there was an effect of sex (*F*(2,68) = 8.198, *p* < 0.001), with females spending more time in the more anxiety-provoking center of the open field than males. There was no effect of irradiation (*F*(6,128) = 0.335, *p* = 0.917) or radiation × sex interaction (*F*(6,68) = 0.445, *p* = 0.847).

During the training in the open field containing two identical objects, there were no effects of radiation or sex or the sex × radiation interaction for distance moved or total time spent exploring both objects in the object recognition test when one familiar object was replaced by a novel one. However, while sham-irradiated mice and mice exposed to 21 cGy or 168 cGy of ^4^He ions showed object recognition and spent more time exploring the novel than the familiar object (Figure 2B), mice exposed to 42 cGy of ^4^He ions showed impaired object recognition and spent comparable times exploring the familiar and novel objects (Figure 2B).

### 2.2. Depressive-Like Behavior in the Forced Swim Test and Contextual and Cued Fear Memory

There were no effects of ^4^He ion irradiation on depressive-like behavior in the forced swim test (Supplementary Figure S1). There were also no effects of irradiation on activity levels prior to the first tone (Supplementary Figure S2A), response to the shock, fear learning (freezing during the tone) or between the tone-shock pairing (freezing between tones). There was an overall effect of sex on response to the shock (*F*(1,64) = 19.489, *p* < 0.001), with higher motion levels in males than females (Supplementary Figure S2B). There were no effects of ^4^He ion irradiation on contextual (Supplementary Figure S2C) or cued (Supplementary Figure S2D) fear memory.

### 2.3. Passive Avoidance Learning and Memory

**Figure 3:**
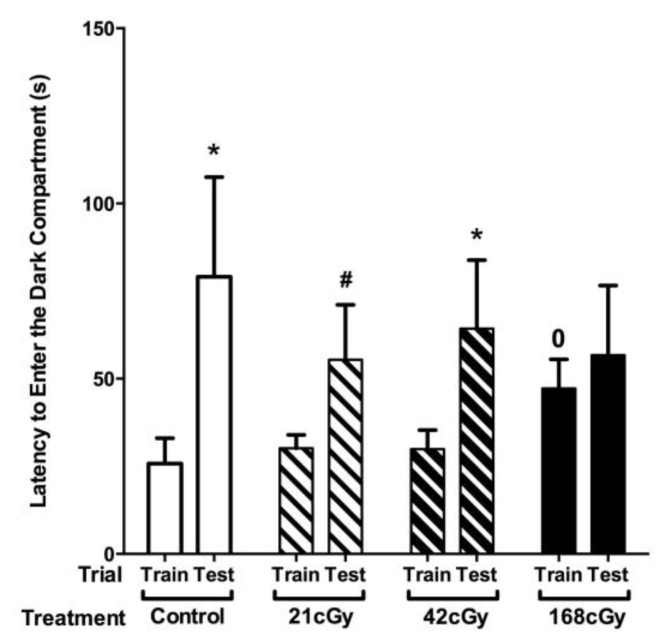
Passive avoidance learning and memory. There were effects of irradiation when the time to enter the dark compartments on Days 1 and 2 were compared using a repeated-measures design within each dose condition. In sham-irradiated mice and mice irradiated with 42 cGy, the latency to enter the dark compartment was significantly higher on Day 2 than Day 1. This did not reach significance in mice irradiated with 21 cGy, and the latency to enter the dark compartment in mice irradiated with 168 cGy was comparable on both days (*p* = 0.27). Control: *n* = 13 mice; 21 cGy: *n* = 23 mice; 42 cGy: *n* = 20 mice; 168 cGy: *n* = 16 mice. * *p* < 0.05; ^#^ *p* = 0.078; ^0^ *p* = 0.0693.

There was no significant effect of ^4^He ion exposure on passive avoidance learning (Figure 3, training). While there was no overall effect of radiation on latency to re-enter the dark compartment during the passive avoidance memory test 24 h later, there were effects of irradiation when the times to enter the dark compartments on Days 1 and 2 were compared using a repeated-measures design within each dose condition (Figure 3). In sham-irradiated mice and mice irradiated with 42 cGy, the latency to enter the dark compartment was significantly higher on Day 2 than Day 1 (*p* < 0.05). In mice irradiated with 21 cGy, this did not reach significance (*p* = 0.078). However, this seems a statistical argument only and might be related to power limitations, as there seemed little if any difference between the performance of mice irradiated with 21 and 42 cGy, while both doses were different than mice irradiated with 168 cGy. In mice irradiated with 168 cGy, the latency to enter the dark compartment was comparable on both days (*p* = 0.27). Increased latency of mice irradiated with 168 cGy during training on Day 1 appears to have contributed to this effect.

### 2.4. MAP-2, CD68, BDNF and ApoE Levels in the Cortex and Hippocampus

**Figure 4:**
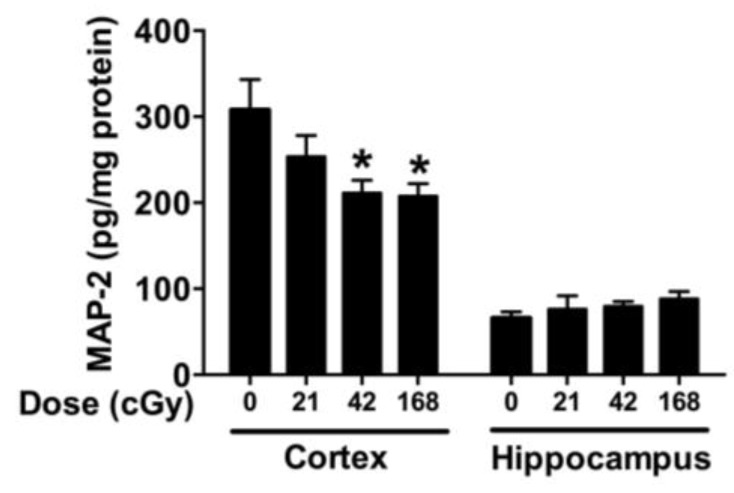
MAP-2 levels in the cortex and hippocampus. There was a radiation × brain region interaction (*F*(3,64) = 4.577, *p* = 0.0058). When the cortex and hippocampus were analyzed separately, in the cortex, there was an effect of irradiation (*F*(3,32) = 3.827, *p* = 0.0189, ANOVA) with mice irradiated with 42 or 168 cGy showing lower cortical level of MAP-2 than sham-irradiated mice. In the hippocampus, there was an effect of irradiation (*F*(3,32) = 4.395, *p* = 0.01, Brown–Forsythe test), but none of the post hoc tests reached significance. *n* = 9 mice/radiation condition/brain region. * *p* < 0.05.

There was a radiation × brain region interaction for the levels of MAP-2 measured (*F*(3,64) = 4.577, *p* = 0.0058). Therefore, the cortex and hippocampus were analyzed separately. In the cortex, there was an effect of irradiation (*F*(3,32) = 3.827, *p* = 0.0189, ANOVA). Mice irradiated with 42 or 168 cGy showed lower cortical level of MAP-2 than sham-irradiated mice (*p* < 0.05, Dunnett’s, Figure 4). There was no effect of sex or sex × radiation interaction. In the hippocampus, there was an effect of irradiation (*F*(3,32) = 4.395, *p* = 0.01, Brown–Forsythe test), but none of the post hoc tests reached significance (Figure 4).

In females, but not males, there was a trend toward higher cortical levels of CD68, a marker of activated microglia, in mice irradiated with 168 cGy (48.5 ± 5.6, *n* = 4 mice) than sham-irradiated mice (67.0 ± 4.3, *n* = 6 mice), but this did not reach significance (*p* = 0.07). There were no effects of irradiation on cortical levels of BDNF in either sex at any of the doses queried. It is conceivable that analysis of mRNA levels might have revealed the effects of irradiation on the transcription of these markers.

**Figure 5:**
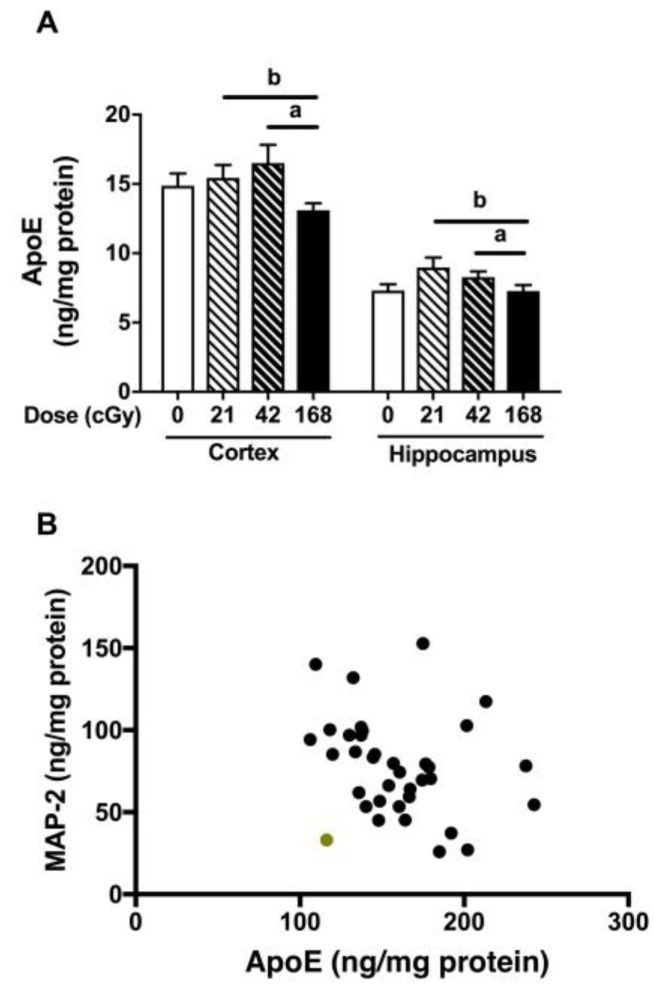
(**A**) ApoE levels in the cortex and hippocampus. There was an effect of irradiation on apoE levels (*F*(3,32) = 3.810, *p* = 0.019, Figure 5A). ApoE levels were higher in mice irradiated with 42 than 168 cGy (*p* = 0.035, Bonferroni’s correction for multiple comparisons, indicated by “a” in the figure), and there was a trend towards higher apoE levels in mice irradiated at 21 than 168 cGy (*p* = 0.065, Bonferroni’s correction for multiple comparisons, indicated by “b” in the figure). (**B**) Relationship between hippocampal MAP-2 and apoE levels (*r* = −0.3277, *p* = 0.0511, two-tailed Spearman correlation, 36 data points). Inspecting the data, there was one data point that seemed removed from the other ones (indicated in green). Therefore, we also performed the analysis without that data point included. The analysis without that data point revealed: *r* = −0.4123, *p* = 0.0138, Spearman correlation, 35 data points.

There was an effect of irradiation on apoE levels (*F*(3,32) = 3.810, *p* = 0.019, Figure 5A). ApoE levels were higher in mice irradiated with 42 than 168 cGy (*p* = 0.035, Bonferroni’s correction for multiple comparisons), and there was a trend towards higher apoE levels in mice irradiated at 21 than 168 cGy (*p* = 0.065). In addition, there was an effect of brain region, with higher apoE levels in the cortex than hippocampus (*F*(1,32) = 153.967, *p* < 0.0001. Finally, there was a trend towards a negative correlation between MAP-2 and apoE levels in the hippocampus (*r* = −0.3277, *p* = 0.0511, two-tailed Spearman correlation, 36 data points, Figure 5B).

## 3. Discussion

^4^He ions are among the most abundant charged particles in the galactic cosmic radiation, and they will represent a significant component of the local fields that astronauts find themselves in during prolonged space missions, such as a mission to Mars [49].

The results of the current study show that the dose of ^4^He ion exposure affecting cognitive performance is test dependent and that different doses affect distinct cognitive functions. For example, while mice irradiated with ^4^He ions at a dose of 42 cGy showed impaired object recognition, in the passive avoidance test, mice irradiated with 168 cGy showed the effect of exposure, with a comparable latency to enter the dark compartment on both days. Therefore, it is important to include multiple cognitive tests as the sensitive dose might depend on the functional outcome measure(s).

The dose-dependent effects of ^4^He ion exposure on object recognition highlight the complexity of determining the risk of cognitive injury. While a dose of 42 cGy impaired object recognition, neither a lower (21 cGy) or higher (168 cGy) dose did so. As recognition of novelty in the environment is critical for astronauts during a space mission and is a function that can easily be tested in humans, as well [50], this impairment is important in assessing the risk of cognitive injury. Future efforts are warranted to determine whether the dose-dependent effects of ^4^He ion exposure on object recognition are associated with alterations in DNA methylation, as we reported for ^56^Fe ion irradiation, another important component of the galactic cosmic radiation environment [27]. 

In contrast to the results of this study with mice in which we saw impairments in novel object recognition without alterations in measures of anxiety in the open field, much lower doses of ^4^He (0.5–10 cGy, 1000 MeV/n) increased measures of anxiety in the elevated plus maze in rats, but did not impair novel object recognition [24]. These data highlight the need to assess the effects of space radiation on measures of anxiety in rats and mice and under various radiation conditions. This is also important as astronauts often participate in multiple missions and receive a cumulative exposure much larger than calculated for a single extended deep space mission (anticipated exposure up to 30 cGy of heavy particles and protons).

In this study, the effects of ^4^He ion irradiation on emotional learning and memory in mice were seen in the passive avoidance test, but not in the fear conditioning test. This could relate to the fact that the fear conditioning paradigm involves unavoidable aversive stimuli, while the passive avoidance test involves a conflict scenario with light and shock as different and avoidable aversive stimuli. The relationship between fear and avoidance behavior is complex [51]. In humans, this relationship was studied by combining Pavlovian differential fear conditioning with a novel task for quantifying spontaneous passive avoidant behavior during self-guided navigation in a virtual reality environment following de novo fear conditioning [52]. The study participants kept their distance from the feared object; the avoidant behavior was not related to the acquisition of fear, but was related to a maladaptive fear expression during extinction training as assessed using fear-potentiated startle. Based on these results, it is conceivable that mice irradiated with ^4^He ions at 168 cGy might show impaired extinction of contextual fear memory. We recognize that while the mice received one shock during passive avoidance training, they received five shocks during contextual and cued fear conditioning. Therefore, it is conceivable that the effects of ^4^He ion irradiation on fear learning and contextual and/or cued fear memory are revealed if only one tone-shock pairing is used. Future studies are warranted to assess these possibilities.

Remarkably, other types of space radiation, including ^56^Fe ions [7,28] and ^28^Si ions [12,13], affected contextual fear memory, while ^16^O ion irradiation affected cued fear memory [17] and ^40^Ca ion exposure performance during fear conditioning training without affecting contextual fear memory [16]. Thus, different components of the space environment affect distinct behavioral and cognitive measures. An open question is how combined exposures, relevant for modeling exposure to astronauts during space missions, might affect behavioral and cognitive performance. This indicates the need for assessing sequential exposures to multiple accelerated ion species in ground-based experiments as well in determining CNS risk following exposure to actual space radiation.

As MAP-2 levels in cognitive brain areas were altered following irradiation in other studies [34,35], we also assessed the effects of ^4^He ion irradiation on hippocampal and cortical MAP-2 levels in the present study. In the cortex, there was a profound reduction in MAP-2 levels following ^4^He ion irradiation at 42 or 168 cGy. As impairments in object recognition were seen at only 21 cGy and altered performance in the passive avoidance test at only 168 cGy, these data suggest that while the MAP-2 levels might have contributed to the alterations in performance in the passive avoidance test, they do not seem required for impairments in object recognition or sufficient for impairments in passive avoidance learning and memory. Consistent with what we reported in ^56^Fe ion-irradiated apoE3 mice, ^4^He ion irradiation significantly affected apoE levels in the hippocampus and cortex. The higher hippocampal and cortical apoE levels in mice irradiated at 42 than 168 cGy might have served as a compensatory protective response preserving their passive avoidance memory. In the hippocampus, there was an effect of irradiation, and although the post hoc test did not reveal significance, the overall radiation effect suggested increased MAP-2 levels following irradiation. This might be due to a compensatory response; increases in hippocampal MAP-2 levels were seen in aged C57BL/6J mice that showed impairments in hippocampus- and cortex-dependent cognitive tests [31] and in aged Rhesus macaques in the hippocampus and prefrontal cortex [33]. The MAP-2 data indicate that at least in the context of ^4^He ion irradiation, the cortex might be more susceptible to injury than the hippocampus. As both brain areas are important for object recognition [50,53,54,55] and passive avoidance learning and memory [56,57,58], increased efforts are warranted to assess and compare the effects of space irradiation on these distinct brain regions. No significant effect of ^4^He ion irradiation was seen on cortical levels of CD68. In contrast, an increase in activated microglia, assessed as immunoreactive ED-1 cells, was seen in the medial prefrontal cortex of male transgenic (Thy1-EGFP) MJrsJ mice 15 and 20 weeks following ^16^O (600 MeV/n; 5 or 30 cGy) and 48Ti (600 MeV/n; 5 or 30 cGy) ion irradiation at six months of age [46]. Differences in the particles and energies used for irradiation, strains of the mice, brain area analyzed and time interval between exposure and analysis might have contributed to these divergent findings.

In summary, the results of the present study show detrimental effects of ^4^He ion irradiation on object recognition and passive avoidance learning and memory and MAP-2 levels in the cortex of male B6D2F1 mice, with non-linear dose-responses, as we and others have reported [59]. We speculate that such non-linear dose-responses may indicate the ability of regions of the brain to elicit compensatory responses when there is a higher level of insult. Other tests performed in the present study, including the forced swim tests and contextual and cued fear learning and memory tests, showed no influence of He ions on the performance at the doses administered. The behavioral and cognitive measures used in this study are relevant, as novel and aversive environmental stimuli are pertinent to conditions experienced by astronauts during and following space missions. Together with earlier studies, these data support the effects of space irradiation on dendritic function. Future studies are warranted to determine the mechanisms underlying these effects and to compare these effects with similar exposures in mice on a different genetic background. Going forward, it will be important to consider exposure conditions that are more relevant to space missions, as the high dose-rate, single acute exposures used in the present study differ substantially to the more chronic, low dose-rate exposures in space. 

## 4. Materials and Methods

### 4.1. Animals and Study Design

The experimental B6D2F1 mice were bred in Dr. Turker’s laboratory to be heterozygous for the selectable adenine phosphoribosyltransferase (*Aprt*) locus on the C57BL/6 background. These mice were obtained by breeding C57BL/6 *Aprt^+/−^* mice with DBA/2 mice. The breeder DBA/2 animals for this study were obtained from Jackson Laboratories (Jax Labs), Bar Harbor, Maine. The C57BL/6 *Aprt^+/−^* strain has been maintained in Dr. Turker’s laboratory for 21 years, with twice-yearly breedings of male C57BL/6 *Aprt^+/−^* to female C57BL/6 mice obtained from Jax Labs at 8 weeks of age. Mice that are heterozygous for *Aprt* have no known phenotype, and even mice that are homozygous deficient for both *Aprt* and hypoxanthine-guanine phosphoribosyl-transferase (HPRT) and devoid of any purine salvage pathways do not show the behavioral alterations seen in Lesch–Nyhan syndrome [60]. To obtain the B6D2F1 mice, we bred 3–4-month-old C57BL/6 *Aprt^+/−^* mice (mostly males, but also some females) with DBA/2 mice obtained from Jax at 8 weeks of age. Breeding cages contained two female mice and one male mouse, and the husbandry procedures were consistent with Jax Labs recommendations. In addition to the work described here, the B6D2F1 mice were also used in an ongoing mutagenesis study funded by NASA. The doses chosen for the ^4^He ion exposures were driven by the requirements of the mutagenesis study.

The experimental mice were 4–6-month-old B6D2F1 female and male mice (*n* = 72 mice in total: sham-irradiation or 250 MeV/n ^4^He ions; LET = 1.6 keV/μm); 0 Gy (*n* = 13 mice; 6 female and 7 male mice); 21 cGy (*n* = 23 mice; 9 female and 14 male mice), 42 cGy (*n* = 20 mice; 11 female and 9 male mice), 168 cGy (*n* = 16 mice; 11 female and 5 male mice). The mice were shipped from Oregon Health & Science University (OHSU) to Brookhaven National Laboratory (BNL) and allowed to accommodate to the housing facility there for one week prior to irradiation at the NASA Space Radiation Laboratory (NSRL). The mice were randomly assigned to the treatment groups described above and were housed under a constant 12 h light: 12 h dark cycle. Food (PicoLab Rodent Diet 20, No. 5053; PMI Nutrition International, St. Louis, MO, USA) and water were provided ad libitum. All procedures were approved by Institutional Animal Care and Use Committees and existing protocols at OHSU and BNL (#IP00001474, 2 March 2018; #IP00000262, 19 June 2007; #285, 12 January 2018).

For irradiation, mice were individually loaded into clear Plexiglas holders (dimensions: 1 1/4” in height, 1 1/4” in width and 3 1/4” in length; TAP Plastics #752) with 22 ventilation air holes 1/8” in diameter and placed in a foam fixture with the long axis of the animal (head to toe) perpendicular to the direction of the beam at the NSRL. Three mice were positioned in the beam line for each exposure in a fixed geometry and received whole body exposures in the absence of anesthesia. Sham-irradiated mice were placed into the plastic enclosures for the same amount of time and on the same day and during the same time of day as the irradiated mice, as described [12]. All mice were housed at three mice per cage in ventilated cage racks at BNL and OHSU. Sham-irradiated and irradiated mice were housed in the same room at both institutions.

The mice were exposed to a square beam of approximately 20 × 20 cm. Dose calibration was performed as described [61]. One week following irradiation or sham-irradiation at BNL, the mice were shipped back to OHSU for behavioral and cognitive testing as described in detail below.

### 4.2. Behavioral and Cognitive Testing

All testing was performed during the light phase three months following ^4^He ion irradiation or sham-irradiation, as described [12]. Investigators at OHSU involved with the behavioral and cognitive testing were blinded to the dose levels until after completion of the experiments. All mice in the same cage were tested at the same time. Mice were tested for behavioral and cognitive performance as indicated in Figure 1.

### 4.3. Exploratory Behavior in the Open Field and Object Recognition (Week 1)

The open field consisted of a brightly-lit enclosure (40.6 × 40.6 cm). It contained a white plastic floor and clear Plexiglas walls (Kinder Scientific, Poway, CA, USA). On three consecutive days, mice were allowed to explore the enclosure for 5 min. Behavioral performance was tracked and analyzed with video software from Noldus Information Technologies set at 15 samples per second (Ethovision XT 7.1, Wageningen, The Netherlands). To analyze exploratory behavior in the open field, distance moved and percent of time spent in the more anxiety-provoking center of the enclosure were analyzed as outcome measures. After each assessment of open-field activity, the equipment was cleaned with 0.5% acetic acid to remove residual odors.

The object recognition test relies on the natural curiosity and propensity of rodents to orient their attention toward a novel stimulus and is sensitive to hippocampal injury [50,62]. Following three days of habituation to the open field, mice were paced into the open field enclosure on Day 4, identical to the procedure described above, except containing two identical objects (small orange hexagonal prisms) placed 15 cm from the adjacent walls and 10 cm apart. The mice were allowed 15 min to explore objects. The next day, one of the familiar objects was replaced with a novel object of identical dimensions. Movement and exploration were tracked and analyzed for nose, body and tail with the video tracking software described above. Nose point location within the object zone was used to determine exploratory behavior. The total time spent exploring both objects was analyzed, and the percent of this total time exploring the novel versus the familiar object was calculated to determine object recognition.

### 4.4. Depression-Like Behavior and Contextual and Cued Fear Learning and Memory (Week 2)

Hippocampus-dependent depression-like behavior was assessed using the forced swim test [63]. In this test, the degree of learned helplessness or behavioral despair, as determined by the scoring of active (swimming and climbing) versus passive (immobility) behaviors in an inescapable cylinder filled with water, is analyzed [64]. Mice were placed in cylinders filled with water (24 °C to a depth of 15 cm) for 6 min, and their behavioral performance was recorded. Following one minute of habituation, the behavioral performance of mice was scored as either immobile or mobile every 5 s for 5 min. The mice were considered immobile once three paws were immobile and the fourth paw exhibited only minimal movement. Behavioral despair was analyzed as the percent of time spent immobile.

Next, mice were tested for contextual and cued fear learning and memory. Mice were trained to associate the environmental context or a discrete cue-tone with a mild food shock, preceded by a short habituation period during which baseline performance was assessed. Contextual fear conditioning is hippocampus and amygdala dependent, while fear conditioning to a cue is amygdala dependent, but hippocampus independent [65,66]. Post-exposure freezing, somatomotor immobility with the exception of respiration, is considered a post-exposure fear response and used as an indicator of conditioned fear. Mice were trained and tested using a Med Associates mouse fear conditioning system (PMED-VFC-NIR-M, Med Associates, St. Albans, VT, USA) utilizing Med Associates VideoFreeze automated scoring system. Mice were placed inside a brightly lit fear conditioning chamber (100 lux) and allowed to habituate for 90 s. Subsequently, the mice were exposed to a 30-s (80 dB at 2800 Hz) tone (cue) paired with a 2-s 0.7-mA foot shock administered at 118 s, co-terminating with the tone at 120 s. This was repeated five times. Twenty four hours later, contextual associative memory was assessed during re-exposure to the training environment for 300 s. Three hours later, mice were exposed to a modified environment (scented with vanilla extract, novel floor texture covering the shock-grid and a black plastic triangular insert for the walls). The mice were allowed to habituate to it for 90 s and exposed to the sound cue for a period of 180 s. Associative learning was analyzed as the percent of time spent freezing in response to the contextual environment or tone. Motion during shock was measured to assess sensor-motor differences in response to the aversive stimulus. During training and assessment of contextual fear memory, the enclosure was cleaned with 0.5% acetic acid between mice. During assessment of cued fear memory, the enclosure was cleaned with 10% isopropanol.

### 4.5. Passive Avoidance Learning and Memory (Week 3)

Hippocampus-dependent contextual fear memory involves re-exposure to an environment in which an aversive stimulus is received during previous training [66]. In the hippocampus-dependent passive avoidance test, the mouse is placed in a light compartment of a two-chamber enclosure, and it receives a foot shock when entering the less anxiety-provoking dark compartment [56,57]. The next day, the mouse is placed back in the lighted compartment, and latency to enter the dark compartment is assessed. Both cognitive tests involve fear learning and memory, but the passive avoidance test involves a conflict scenario (aversive light versus memory of the shock). To assess performance in the passive avoidance test, mice were placed in a brightly-lit compartment of a chamber also containing a dark compartment (Hamilton-Kinder, Poway, CA, USA). After 5 s of acclimation, the bright house light turned, on and a connecting gate to the dark compartment opened. The mouse, preferring the darkened left side, typically steps quickly through the gate to enter the dark compartment. Subsequently, the mouse receives a brief and slight foot shock (0.35 mA for 3 s). Each mouse was given a trial of up to 120 s without entering the dark compartment. After 24 h, the mouse was again placed in the right compartment. After a 5-s period, the light of the compartment switched on, and the connecting door opened; and the time to re-enter the dark compartment was measured for up to 300 s.

### 4.6. MAP-2, CD68, BDNF and ApoE ELISAs

For assessments of MAP-2, CD68 and BDNF levels, the mice were killed and the hippocampus and cortex in their brains dissected out. The hippocampus and cortex were homogenized and a protein assay performed using a BCA kit (Fisher Scientific, Chicago, IL, USA) as described using a MyBiosource MAP-2, CD68, BDNF or apoE ELISA (San Diego, CA, USA) and following the assay instructions. The standard curve was run in duplicate and the samples as single samples. There were 9 samples per radiation condition per brain region. Based on the optical density values, the MAP-2, CD68, BDNF and apoE levels in the samples were calculated using GraphPad Prism software (San Diego, CA, USA).

### 4.7. Statistical Analyses

All data are shown as the mean ± the standard error of the mean (SEM). Statistical analyses were performed using SPSS™ (version 22, Chicago, IL, USA) software packages. The data from the training day were analyzed using ANOVAs with radiation and sex as between group factors, followed by post hoc tests when appropriate. When sex was not a significant factor, it was dropped from the model. Performance over multiple trials was analyzed by repeated-measures ANOVA. If violation of sphericity occurred indicating that the variances of the differences between all combinations of the groups were not equal, Greenhouse–Geisser corrections were used. Sidak’s post hoc tests were used. All figures were generated using GraphPad Prism software (San Diego, CA, USA). We considered *p* < 0.05 as statistically significant.

## Supplementary Materials

The following are available online at http://www.mdpi.com/1422-0067/19/4/1247/s1.
