# Supplementary material for: Detrimental Effects of Helium Ion Irradiation on Cognitive Performance and Cortical Levels of MAP-2 in B6D2F1 Mice
Source: Int J Mol Sci. 2018 Apr 20;19(4):1247. doi: 10.3390/ijms19041247 (PMC5979430; doi:10.3390/ijms19041247)
Supplement: Supplementary file 1 [file ijms-19-01247-s001.pdf]

International Journal of
Molecular Sciences

MDPI

Supplementary Figure Legends

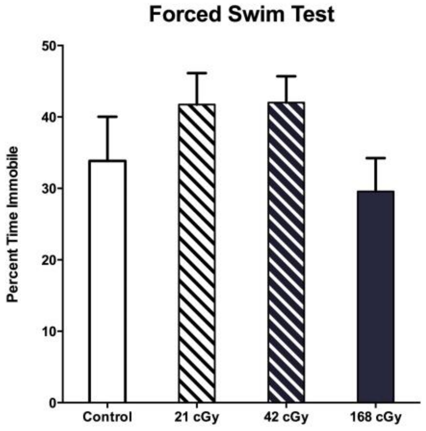

Figure S1. No significant effects of ^{4}He ion irradiation on the percent time spent immobile in the forced swim test. Control: n = 13 mice; 21 cGy: n = 23 mice; 42 cGy: n = 20 mice; 168 cGy: n = 16 mice.

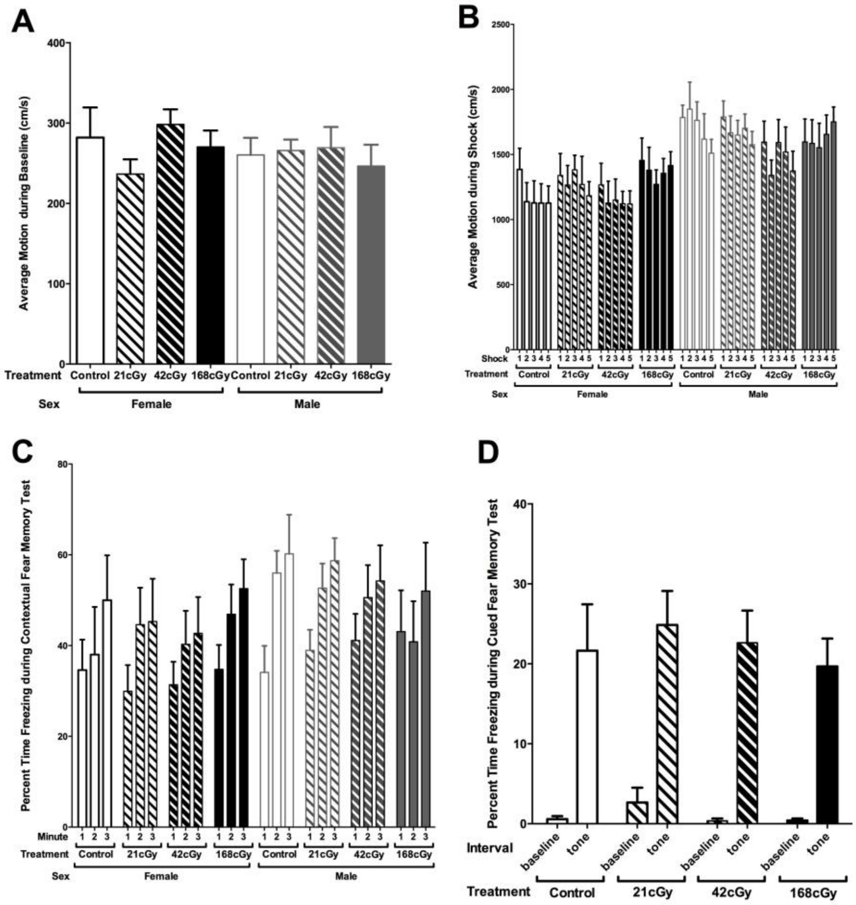

Figure S2. No significant effects of ^{4}He ion irradiation on contextual or cued fear learning or memory. **A.** No effects of Helium ion irradiation on activity levels prior to the first tone. **B.** No effect of Helium ion irradiation on response to the shock. There was an effect of sex on response to the shock ($F(1,64) = 19.489$, $p < 0.001$), with higher motion levels in males than females. **C.** No effects of He ion irradiation on contextual fear memory. **D.** No effects of Helium ion irradiation on cued fear memory. Control: n = 13 mice; 21 cGy: n = 23 mice; 42 cGy: n = 20 mice; 168 cGy: n = 16 mice.
